# B7-H4 as an independent prognostic indicator of cancer patients: a meta-analysis

**DOI:** 10.18632/oncotarget.18566

**Published:** 2017-06-19

**Authors:** Zibo Meng, Feiyang Wang, Yushun Zhang, Shoukang Li, Heshui Wu

**Affiliations:** ^1^ Department of Pancreatic Surgery, Union Hospital, Tongji Medical College, Huazhong University of Science and Technology, Wuhan 430022, People's Republic of China

**Keywords:** B7-H4, cancer, prognosis, meta-analysis

## Abstract

The expression of B7-H4 was observed in a variety of tumors, however the prognostic value in cancer was still controversial. Therefore, we conducted this meta-analysis to explore the potential role of B7-H4 in cancer prognostic prediction. Twenty-seven studies including 3771 patients were brought into the analysis according to the inclusion and exclusion criteria. The pooled results demonstrated that elevated B7-H4 predicted a poor OS (HR = 1.93, 95% CI 1.71-2.18, *P* < 0.001) and DFS (HR = 1.84, 95% CI 1.46-2.33, *P* < 0.001). Subgroup analysis showed that races, tumor types, sample sources, analysis types, sources of HR and sample sizes exhibited non-significant distinctions with OS (P_S_ = 0.878, P_S_ = 0.143, P_S_ = 0.613, P_S_ = 0.639, P_S_ = 0.48 and P_S_ = 0.528, respectively). PubMed, Embase and the Cochrane Library were searched up to April 7, 2017, to recognize the available studies for assessing the association between B7-H4 and cancer patients’ outcome. We extracted the hazard ratio (HR), relative ratio (RR), odds ratio (OR) with their 95% confidence interval (CI) for overall survival (OS) or disease-free survival (DFS) as the effect size (ES) for the analysis. This meta-analysis demonstrates high expression of B7-H4 is a negative correlation with the outcome of cancer patients.

## INTRODUCTION

According to GLOBOCAN 2012 report [1], there were about 14.1 million new cancer cases and 8.2 million deaths related to the cancer in 2012. By 2025, it predicted that there would be 19.3 million new cases of cancer per year. In America, cancer is the second leading cause of death and promises to be the first place for the cause of death in next few years [2]. In spite of that, a large number of researchers are involved in cancer therapeutic and diagnostic works, the prognosis is still poor for most malignancies. Therefore, seeking prognostic indicators for the cancer seem important in our future scientific research.

In the past few years, B7-H4 (B7S1/VTCN1/B7x), a member of B7/CD28 superfamily, has been studied in many kinds of malignant tumors, including breast, lung, gastric, pancreatic, colorectal, melanoma and prostate cancer. Besides, the expression level of B7-H4 in these cancer patients is obviously extensive compared with normal ones [3]. Recent studies have shown that B7-H4 can inhibit the proliferation, activation and cytokine secretion of T cells in tumor environment, and the B7-H4 expression is associated with many clinicopathological parameters such as tumor size, primary tumor classification, TNM malignant tumor score, overall survival, and tumor infiltrating T cells [3, 4]. Recently, cancer therapies with antibodies against PD-L1 were shown to increase lifetime of particular sorts of cancers. However, not all of the cancer patients would respond to these drugs [5–7]. PD-L1 is expressed in some human cancers, but as described above, B7-H4 is expressed in a much wider range of tumors [8]. Therefore, considered the more extensive expression of B7-H4 compared with the PD-L1 in human cancer and the mechanism of B7-H4 regulating tumor development and progression, we suspect that B7-H4 may be used as a potential target for cancer treatment.

Several literatures have reported that decreased overall survival (OS) and disease-free survival (DFS) is related to the overexpression of B7-H4 [9–11]. However, Zang et al reported that B7-H4 overexpression was not notably associated with DFS (*p* = 0.1) [12, 13]. Moreover, B7-H4 can be detected both in tumor tissue and patients’ blood, and there still lacks a systematic pooled analysis of these two aspects so far. Thus, we conducted this meta-analysis to assess the role of B7-H4 in the prognosis of patients with cancer.

## RESULTS

### Excluded and included studies

**Figure 1:**
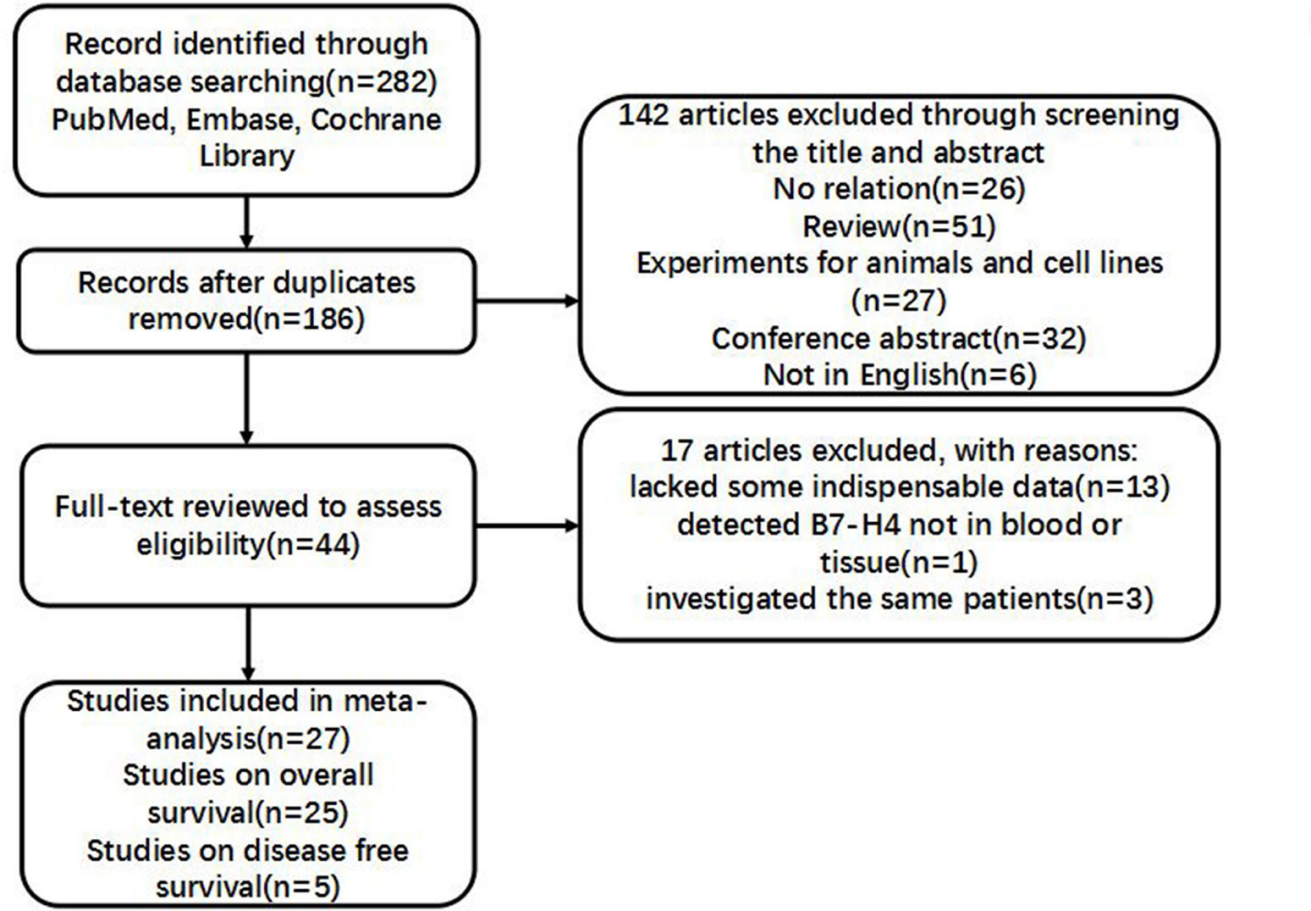
Flow chart for research selection strategy

A total of 282 articles were initially found by using above-mentioned searching strategy. After a screening of the titles or abstracts, 44 records were identified associated with the relationship between the expression of B7-H4 and patient prognosis in kinds of malignant tumors. Among these, 17 articles were excluded (13 lacked important data, 1 detected B7-H4 not in tissue or blood and 3 covered the same patient population). As a result, 27 studies were enrolled for present meta-analysis (Figure 1).

### Study characteristics

In total, 3771 patients from China, Japan, Korea, Canada, America, Germany and Greece were contained in these eligible studies and Table 1 exhibited the major clinical characteristics, and Supplementary Table 2 showed other characteristics in detail. These enrolled patients were diagnosed with a variety of cancers, including cervical cancer, pancreatic cancer, oral squamous cell carcinoma, lung cancer, esophageal carcinoma, renal cell carcinoma, hepatocellular carcinoma, gastric cancer, osteosarcoma, urothelial cell carcinoma, thyroid cancer, melanoma, esophageal squamous cell carcinoma, ovarian cancer, prostate cancer. Seventeen studies (62.9%) were published in 2014 or later. The majority of studies (81.5%) reported on Asians, and five studies (18.5%) reported on Caucasians. Various cut-off values were shown in these literatures. B7-H4 were detected in tissue and blood, as each was expressed in 22 and 6 articles. The endpoints OS and DFS were respectively addressed in 25 and 5 studies. Twenty-two studies directly reported the HRs or RRs or ORs and the other articles’ parameters were estimated through survival curves indirectly.

**Table 1:** Characteristics of studies included in the meta-analysis

Study	Year	Country	Tumor type	Sample size	B7-H4 source	Detected method	B7H4 (+/−)	Multivariate analysis	Hazard Ratio provided (SC/Report)	Outcome measures (OS/DFS)
Krambeck [35]	2006	USA	RCC	259	Tissue	IHC	153/106	Yes	Report	OS
Wang [11]	2016	China	Lung cancer	316	Blood	ELISA	224/92	Yes	Report	OS
Liang [36]	2013	China	Colorectal cancer	185	Tissue	IHC	117/68	No	Report	OS/DFS
Fukuda [37]	2016	Japan	RCC	181	Blood	ELISA	90/91	Yes	Report	OS
Wu [38]	2016	China	OSCC	165	Tissue	IHC	83/82	No	Report	OS
Jiang [39]	2010	China	GC	156	Tissue	IHC	70/86	Yes	Report	OS/DFS
Shi [40]	2014	China	GC	132	Blood	ELISA	66/66	Yes	Report	OS
Arigami [41]	2011	Japan	GC	120	Tissue	IHC	31/89	Yes	Report	OS
Zhang [42]	2015	China	Hepatocellular carcinoma	116	Blood	ELISA	64/52	Yes	Report	OS
Chen [43]	2011	China	Esophageal carcinoma	112	Tissue	IHC	66/46	No	Report	OS
Huang [44]	2016	China	Cervical cancer	108	Tissue	IHC	87/21	Yes	Report	OS
Dong ^a,^ [45]	2015	China	Osteosarcoma	190	Tissue/blood	IHC/ELISA	73/31; 36/50	Yes	Report	OS
Liu [9]	2014	Japan	Cervical cancer	102	Tissue	IHC	71/31	Yes	Report	OS/DFS
Oikonomopoulou [46]	2008	Canada	Ovarian cancer	98	Blood	ELISA	49/49	Yes	Report	OS
Zhang [14]	2015	China	Hepatocellular carcinoma	93	Blood	ELISA	32/61	Yes	Report	OS
Wang [47]	2015	China	Esophageal carcinoma	66	Tissue	IHC	48/18	Yes	Report	OS
Zhu [48]	2013	China	Thyroid cancer	64	Tissue	IHC	46/18	No	SC	OS
Chen [30]	2014	China	PC	63	Tissue	IHC	31/32	No	SC	OS
Fan [49]	2014	China	Urothelial cell carcinoma	62	Tissue	IHC	47/15	Yes	Report	OS
Maskey [50]	2014	China	GC	56	Tissue	IHC	12/44	No	SC	OS
Li [51]	2013	China	Lung cancer	49	Tissue	IHC	20/29	Yes	Report	OS
Tsiaousidou [10]	2015	Greece	PC	41	Tissue	IHC	16/25	Yes	Report	OS
Quandt [52]	2011	Germany	Melanoma	29	Tissue	IHC	21/8	No	SC	OS
Zang [13]	2007	USA	Prostate cancer	823	Tissue	IHC	120/694	No	Report	DFS
Jung [53]	2011	Korea	RCC	102	Tissue	IHC	18/84	Yes	SC	DFS
Qian [54]	2016	China	PC	43	Tissue	IHC	28/15	Yes	Report	OS
Xu [12]	2016	China	PC	40	Tissue	IHC	30/10	Yes	Report	OS

RCC: renal cell carcinoma; OSCC: oral squamous cell carcinoma; GC: gastric cancer; PC: pancreatic cancer; IHC: immunohistochemistry; ELISA: enzyme-linked immunosorbent assay; SC: survival curve.

^a^ This article contained two cohorts, one cohort was detected from tissue, and the other was detected from blood.

### Quality assessment

We conducted the quality assessment for each of 27 studies included in our meta-analysis. All studies were scored in accordance with the NOS and the quality score varied from 7 to 9, with a mean of 8.0. For more details, please see Supplementary Table 1. Better methodology could get a higher score and all the literatures were selected in the subsequent calculation.

### Meta-analysis results

### Overall survival

**Figure 2:**
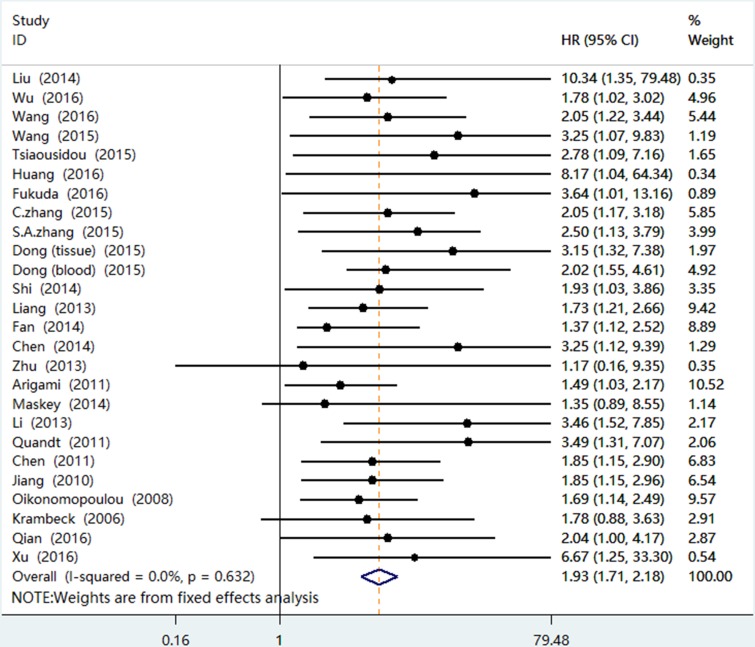
Forest plots of studies evaluating hazard ratios of B7-H4 overexpression in cancer patients

Twenty-five studies with 26 groups of people, including 2846 patients, provided proper information for OS analysis. This meta-analysis major results were shown in Table 2. We used the fixed-effects model to pool HRs because non-significant heterogeneity was found (I^2^ = 0.0%, *P* = 0.632) among studies. The merged HR of 1.93 (95% CI 1.71–2.18, *P <* 0.001) demonstrated that high levels of B7-H4 expression were statistically associated with a worse prognosis (Figure 2). And the forest plots for the association between B7-H4 overexpression and prognosis were shown in Figure 2.

**Table 2:** Pooled hazard ratios for OS based on subgroup analyses

Outcome subgroup	Number of patients	Number of studies	Fixed-effects model			Heterogeneity	
			HR(95%CI)	*P* value	*P*_S_value	I^2^	*P*
Overall survival	2846	25	1.93 (1.71–2.18)	< 0.001		0.0%	0.632
Ethnicity					0.878		
Asian	2419	21	1.92 (1.68–2.19)	< 0.001		0.0%	0.58
Caucasian	427	4	1.97 (1.46–2.66)	< 0.001		0.0%	0.399
Tumor type					0.143		
Renal cell carcinoma	440	2	2.10 (1.13– 3.91)	0.019		0.0%	0.34
Pancreatic cancer	187	4	2.69 (1.66–4.34)	< 0.001		0.0%	0.60
Lung cancer	365	2	2.38 (1.54–3.69)	< 0.001		10.4%	0.29
Hepatocellular carcinoma	209	2	2.22 (1.51– 3.27)	< 0.001		0.0%	0.62
Gastric cancer	464	4	1.65 (1.27–2.14)	< 0.001		0.0%	0.84
Esophageal carcinoma	178	2	2.01 (1.31–3.08)	0.001		0.0%	0.36
Cervical cancer	210	2	9.20 (2.16–39.22)	0.003		0.0%	0.87
Others	793	7	1.78 (1.47–2.14)	< 0.001		0.0%	0.52
Sample source					0.613		
Tissue	1824	19	1.89 (1.63–2.20)	< 0.001		9.0%	0.35
Blood	1022	7	2.00 (1.63–2.46)	< 0.001		0.0%	0.91
Analysis type					0.639		
Multivariate	2210	18	1.94 (1.68–2.23)	< 0.001		1.2%	0.442
Univariate	636	7	1.90 (1.50– 2.41)	< 0.001		0%	0.699
Source of HR					0.638		
SC	460	4	2.53 (1.46–4.38)	0.001		0.0%	0.474
Report	2386	21	1.90 (1.68–2.15)	< 0.001		0.0%	0.61
Sample size					0.528		
> 100	2056	13	1.88 (1.60–2.20)	< 0.001		0.0%	0.70
< 100	790	13	2.01 (1.66–2.42)	< 0.001		6.1%	0.39

**Figure 3:**
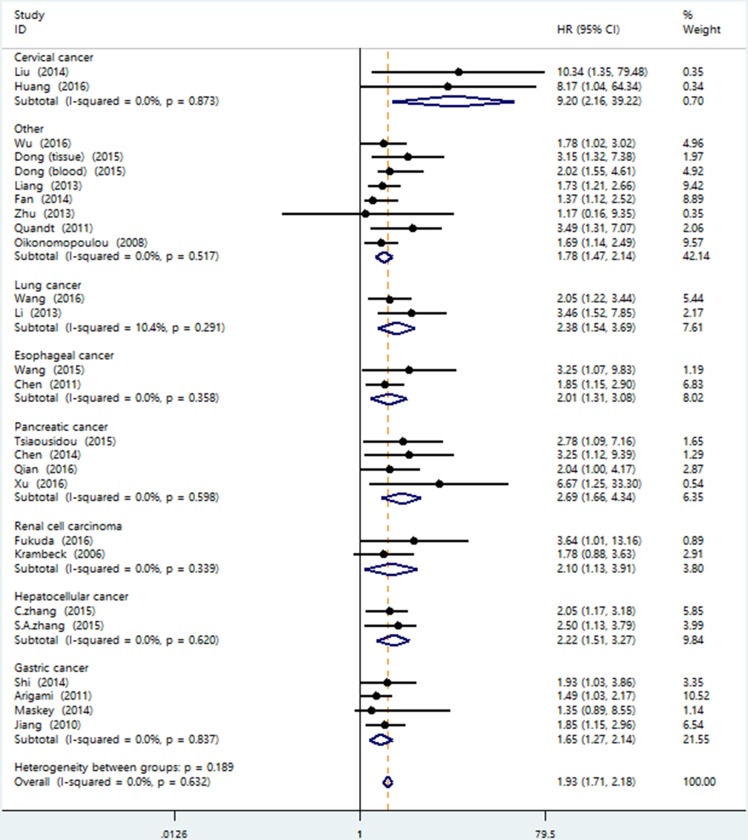
Forest plot of the association between B7-H4 overexpression and OS in a variety kinds of patients

Because of the unremarkable heterogeneity among studies, we conducted the subgroup analysis using the fixed-effects model on the basis of the tumor type, sample source, ethnicity, the source of HR, analysis type and sample size. As we could see in Figure 3, B7-H4 overexpression indicated a worse survival in patients with renal cell carcinoma (pooled HR = 2.10; 95% CI = 1.13–3.91; *P* = 0.019), pancreatic cancer (pooled HR = 2.69; 95% CI = 1.66–4.34; *P <* 0.001), lung cancer (pooled HR = 2.38; 95% CI = 1.54–3.69; *P <* 0.001), hepatocellular carcinoma (pooled HR = 2.22; 95% CI = 1.51–3.27; *P <* 0.001), gastric cancer (pooled HR = 1.65; 95% CI = 1.27–2.14; *P <* 0.001), esophageal carcinoma (pooled HR = 2.01; 95% CI = 1.31–3.08; *P* = 0.001), cervical cancer (pooled HR = 9.20; 95% CI = 2.16–39.22; *P* = 0.003) and others (pooled HR = 1.78; 95% CI = 1.47–2.14; *P <* 0.001). Elevated B7-H4 had negative effects on overall survival in Asian (pooled HR = 1.92; 95% CI = 1.68–2.19; *P <* 0.001) and Caucasian (pooled HR = 1.97; 95% CI = 1.46–2.66; *P <* 0.001) populations. In terms of specimen type, the overexpression of B7-H4 detected from tissue (pooled HR = 1.89; 95% CI = 1.63–2.20; *P <* 0.001) and blood (pooled HR = 2.00; 95%CI = 1.63–2.46; *P <* 0.001) both revealed poor prognosis in cancer patients. Pooled hazard ratio results were all > 1 based on ethnicity, tumor type, sample source, analysis type, source of HR and sample size (Table 2).

**Figure 4:**
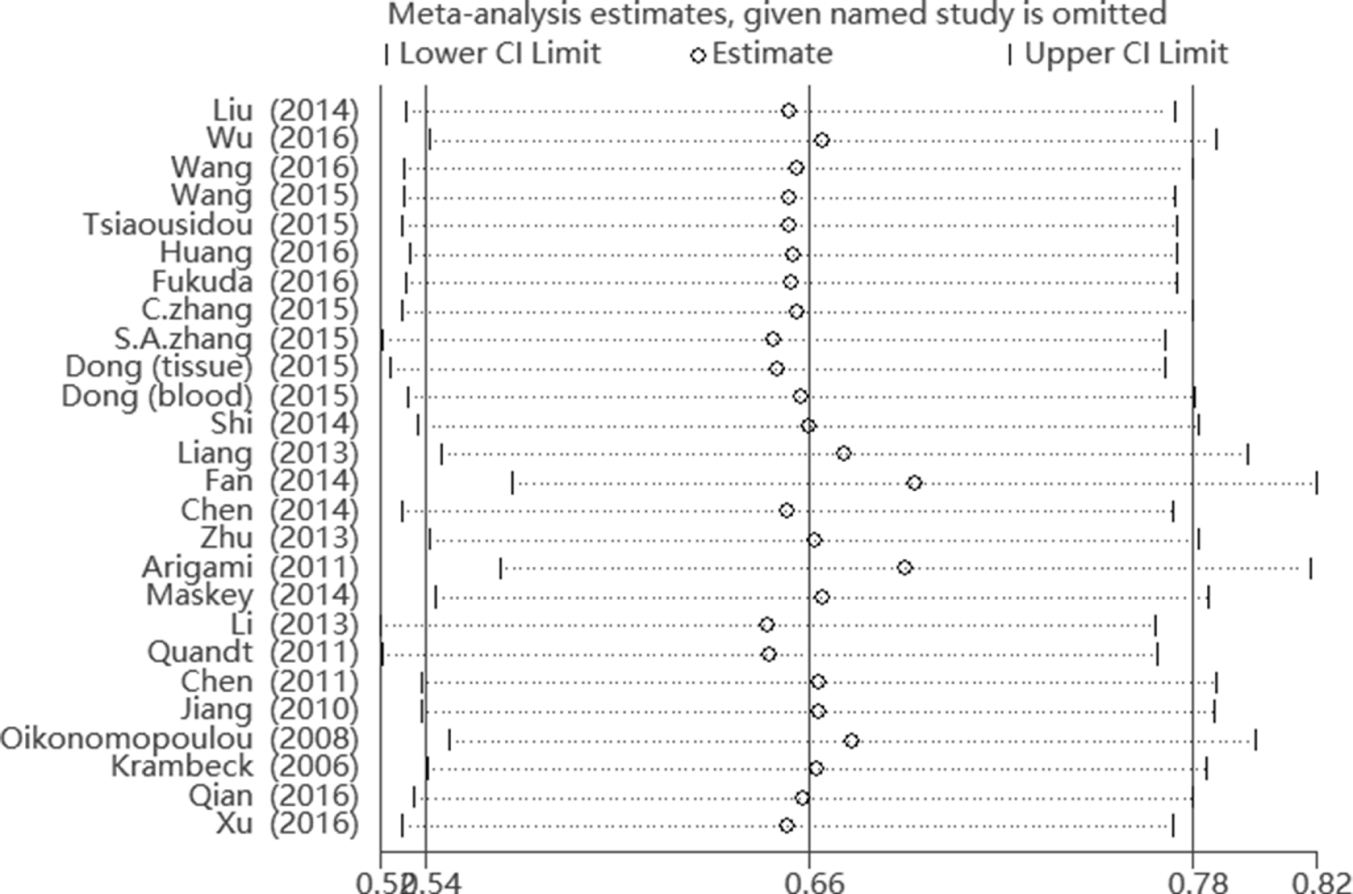
Sensitivity analysis of the meta-analysis of B7-H4 overexpression

We conducted fixed-effects model sensitivity analysis via removing the literature data one by one, and showed no substantial influence. (Figure 4) A meta-regression was applied to assess the reason for the heterogeneity. Ethnicity (*P*_S_ = 0.878), tumor type (*P*_S_ = 0.143), sample size (*P*_S_ = 0.528), sample source (*P*_S_ = 0.613), analysis type (*P*_S_ = 0.639) and source of HR (*P*_S_ = 0.638) were not reasons for the heterogeneity. Multiple elements may contribute to the integral heterogeneity.

### Publication bias

**Figure 5:**
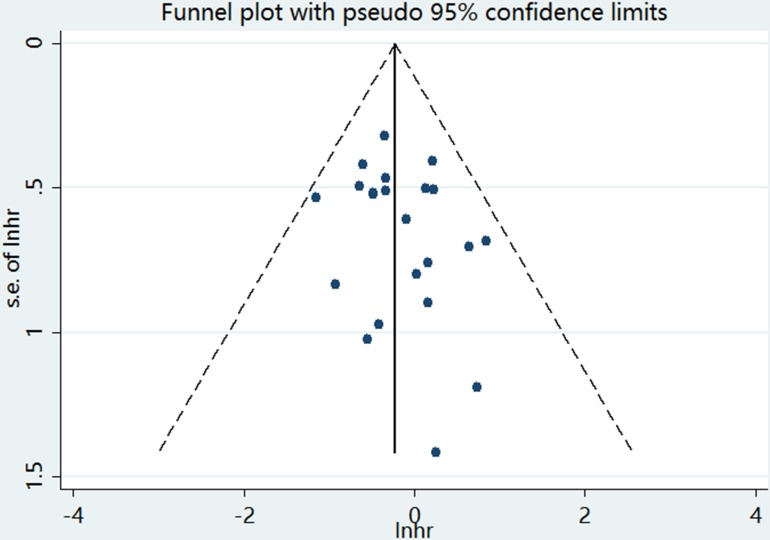
Funnel plot for publication bias assessment

We measured the publication bias of articles through funnel plots, and Egger's and Begg's tests. In Figure 5, asymmetrical funnel plots were shown and reflected significant publication bias. Egger's tests (*P* < 0.001) also proved this conclusion. In order to adjust the publication bias, we conducted a trim and fill analysis under fixed-effects model. Adjusted pooled HR for OS was 1.80 (95% CI = 1.61–2.03).

### Disease free survival

**Figure 6:**
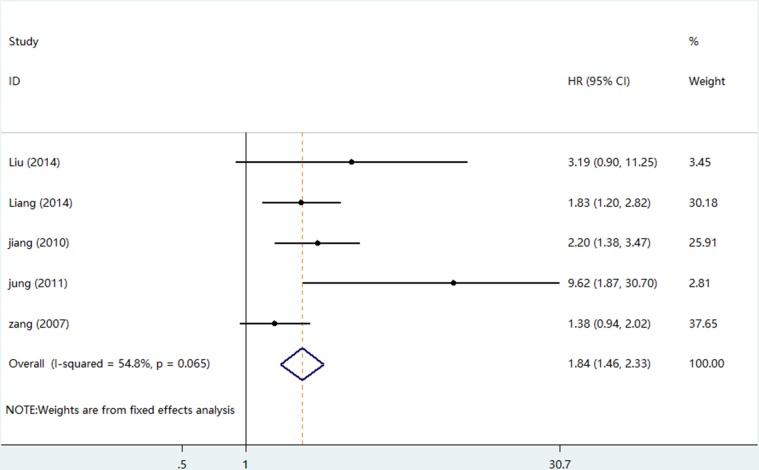
The forest plot of HRs for DFS

Five studies suitable data pooled for DFS analysis which including a number of 1368 patients (Table 3). There was no obvious heterogeneity among the studies (I^2^ = 54.8%, *p* = 0.065), because of this, fixed-effects model was used to pool HR for DFS. Analysis results revealed that higher levels of B7-H4 expression were associated with poorer DFS compared with the lower ones (pooled HR = 1.84; 95%CI = 1.46–2.33; *P <* 0.001). The forest plot of HRs for DFS was shown in Figure 6. Subgroup analysis based on ethnicity revealed B7-H4 induced a worsen DFS in Asian patients (HR = 2.20; 95% CI = 1.63–2.96; *P <* 0.001) than Caucasian patients (HR = 1.38; 95% CI = 0.94–2.02; *P* = 0.099). Overexpression B7-H4 had the worst effect in renal cell carcinoma with the highest pooled HR (HR = 9.62; 95% CI = 2.37–38.98; *P* = 0.002).

**Table 3:** Pooled hazard ratios for DFS based on subgroup analysis

Outcome subgroup	Number of patients	Number of studies	Fixed-effects model			Heterogeneity	
			HR(95%CI)	*P* value	*P*_s_value	I^2^	*P*
Disease free survival	1368	5	1.84 (1.46–2.33)	< 0.001		54.80%	0.065
Ethnicity					0.340		
Asian	545	4	2.20 (1.63–2.96)	< 0.001		43.60%	0.150
Caucasian	823	1	1.38 (0.94–2.02)	0.099			
Tumor type					0.506		
Cervical cancer	102	1	3.19 (0.90–11.28)	0.072			
Colorectal cancer	185	1	1.83 (1.19–2.81)	0.006			
Gastric cancer	156	1	2.20 (1.39–3.49)	0.001			
Renal cell carcinoma	102	1	9.62 (2.37–38.98)	0.002			
Prostate cancer	823	1	1.38 (0.94–2.02)	0.099			

HR = hazard ratio; CI = confidence interval; Ps = P for subgroup difference.

## DISCUSSION

The relationship between B7-H4 overexpression and prognosis in patients with cancer still remained not fully understood. In order to further investigate the connection between clinical outcome and B7-H4 expression, we conduct this review which is the first meta-analysis using the B7-H4 testing results which detected in blood and tissue.

B7-H4 is a newborn member of B7/CD28 family which has been identified in 2003 [4]. In the immune response, B7 superfamily has pivotal effect in regulating process, which is relevant to the initiation, development and recurrence of cancer [14, 15]. The biological mechanism of B7-H4 is mainly participate in tumor immune escape. Studies have shown that expression of B7-H4 within the tumor microenvironment can increase the function and quantity of FOXP3+ regulatory T cells (Treg cells) and facilitate the immune tolerance also infiltration capacity of Treg cells [16, 17]. By this means, Tregs are able to inhibit the proliferation of CD4+/CD8+ T cells and then suppress anti-tumor immune responds [18]. Several studies have shown that B7-H4 expression is negatively associated with the degree of tumor-infiltrating immune cells [19, 20]. It participates in the inhibition of immune cells infiltration, and this mechanism may be one of the reasons for the interpretation of tumor immune escape. Previous studies have been confirmed that the aberrant activation of Erk1/2 and AKT signaling pathway has an important affect in many tumor biological processes. *In vivo* and *in vitro* experiments confirmed that, B7-H4 silence increased the apoptosis of tumor cells, which was associated with the activity of caspase and decreased Erk1/2 and AKT phosphorylation [21, 22]. Zhang et al reported that B7-H4 can promote the G1/S phase transition and then enhance the tumor cell proliferation *in vitro* and tumorigenicity *in vivo* [23]. Another article confirmed that B7-H4 silence suppressed IL-6 secretion through JAK2/STAT3 inactivation and further downregulating cell proliferation [24]. For these reasons above, we believed that B7-H4 overexpression may predict a bad prognosis for cancer patients.

Blocking inhibiting signals for T cells in order to enhance the anti-tumor effects has become a promising cancer therapy, and it has been implemented through CTLA-4 and B7-H1 blockade in early years [5, 25, 26]. However, the CTLA-4 ligand expression location is limited to the antigen presenting cells in lymphoid tissue. The CTLA-4 functions are restricted to the early stage of the immune response. Compared with blocking the B7 family members which express precisely on various tumor, controlling the upstream stages in turn regulating effector T cells acting on peripheral tumors seems inefficient and imprecise [27]. B7-H1 is another potential immunotherapy target which is expressed on tumor and normal tissues, but B7-H4 can hardly be discovered in normal tissue and the expression pattern in tumor is wider than B7-H1[28, 29]. Chen et al. [30] also reported that B7-H4 overexpression was correlated with pancreatic cancer patients’ OS (*P* < 0.001), whereas the expression of B7-H1 didn't (*p* = 0.089). For the reasons above, B7-H4 has the potential to become a new immunotherapy checkpoint for cancer treatment.

In our analysis, the pooled HR of B7-H4 on OS is 1.93 (HR = 1.93, 95% CI 1.71–2.18, *P <* 0.001), and HR = 1.84 on DFS (95% CI = 1.46–2.33; *P <* 0.001) which verified B7-H4 was related to a worse outcome for cancer patients. However, Zang et al. [13] reported that B7-H4 wasn't an independent prognostic indicator, the reason why this phenomenon appeared may rely on the limited follow-up years for the long-survival period of prostate cancer patients. The previous meta-analysis which had investigated the high expression of B7-H4 influenced the prognosis of solid tumor patients also showed this trend as we illustrated [29]. While that article only included the samples derived from the tumor tissue and only contained 18 articles. Previous articles had shown the prognostic value of the blood-derived B7-H4. In addition, the blood-derived B7-H4 obtained by noninvasive methods was more clinically valuable than the tissue-derived B7-H4 obtained by invasive means. It was meaningful to conclude blood-derived B7-H4 to discussion. The present analysis had solved this question, and by subgroup analysis we found that the high expressions of blood-derived B7-H4 and tissue-derived B7-H4 were both independent prognostic indicators for poor OS (HR = 2.00 and HR = 1.89, respectively), also, high expression of B7-H4 in the blood could better reflect the poor prognosis of the disease. Therefore, it is the first meta-analysis on the relationship between B7-H4 detected in tissue/blood and OS/DFS and other clinical parameters.

However, several limitations still exist in our study. First, this meta-analysis was restricted to the published English Literatures, several studies couldn't include because of its linguistic disparities or negative results which couldn't be published. Second, although our analysis contained more articles and supplied the studies with B7-H4 expressed in blood compared with the previous one, the number is still inadequate, and more studies are needed to be included to make the results more credible. Third, the data of several studies were extracted from survival curves, the statistical error was inevitable which could affect the precision of results and the differences could be observed compared with the former article [29]. Fourth, the detection methods and cut-off values were varied, and the inconformity of these items may result in the heterogeneity of overall results. Unified detection methods and division criteria of high expression B7-H4 should be established to make it suitable for clinical applications. Fifth, although we did the subgroup analysis according to the ethnicity, tumor type, sample size, sample source, analysis type and source of HR, there were still other potential influencing factors to explain the variation of expression patterns in the same cancer types. However, due to the methodological reasons, many articles cannot provide the sufficient data (HR and corresponding 95% CI) to do the subgroup analysis on other factors. In our future research, we considered to narrow the scope of study (for example, conducting a single type of tumor study) to explore these differences. Last, the majority of samples were collected from Asian patients especially patients in China, so a wider sample collection is necessary to avoid the selection bias.

## MATERIALS AND METHODS

The present meta-analysis was conducted in accordance with the Meta-analysis of Observational Studies in Epidemiology group (MOOSE) [31].

### Literature search strategy

The relevant articles were systematically selected through PubMed, Embase and the Cochrane Library. We used the following strategies to select studies:” B7-H4 OR V-Set Domain-Containing T-Cell Activation Inhibitor 1 OR B7H4 OR B7x OR VTCN1 OR B7S1” (all fields) AND “Tumor OR Neoplasm OR Neoplasia OR Cancer OR carcinoma OR tumour OR leukemia OR myeloma OR lymphoma OR Malignancy OR Carcinomatosis OR Carcinomatoses” (all fields) AND “prognostic OR prognosis OR prognostication OR Prognoses OR survive OR Survived OR survival OR outcome” (all fields). Two authors (ZB. Meng and SK. Li) independently conducted the search. The last search was carried out on April 7, 2017.

### Inclusion and exclusion criteria

Studies were qualified if they met the following criteria: (a) B7-H4 expressed in tumor tissue or blood; (b) the relationship between B7-H4 expression and tumor prognosis was examined; (c) provided sufficient and effective information to estimate the survival date such as: the hazard ratio (HR) or relative ratio (RR) or odds ratio (OR) with 95% confidence intervals (CI); (d) studies published in English. The literatures were excluded if they were duplicate reports, reviews, letters, conference abstracts, case reports, experimental and animal studies. When several research covered on the same patient cohort, the most recent literature was chosen.

### Data extraction and quality assessment

Relevant studies were screened and required data were extracted independently by two researchers (ZB. Meng and FY. Wang). The following information was collected: first author; publication year; country; cancer type; sample size; gender; tumor stage; follow-up period; B7-H4 source (blood or tissue); detected methods; cut-off value; outcome measures; HR for survival outcome and corresponding 95% CI. If a study provided univariate and multivariate results both, considered the precision of data, the multivariate analysis would be selected. Some HRs and their 95% CI were not directly reported in the articles, we using the graphical data to extract information.

Two reviewers evaluated the quality of studies according to the Newcastle-Ottawa Quality Assessment Scale (NOS) [32]. The quality scores ranged from 0 to 9(9 was highest). The research with scores higher than 6 was considered as high quality.

### Statistical analysis

The analysis was calculated by the STATA version 14.0 (Stata Corporation, College Station, TX, USA). The B7-H4 cut-off values classified cancer patients into high and low expression group. HRs with 95% CI were combined to assess the effective value on prognosis. Because the outcome of tumor is rare in all population and subgroups under review, the odds ratio(OR), rate ratio(RR), and hazard ratio(HR) can generally ignore the distinctions [33], the pooled ORs or RRs with 95%CI were appropriate for the assessment. If the above statistical variables were provided in the article, we used them directly. We also contacted the authors of the literature to obtain the relevant data. The rest of articles’ information we read the Kaplan-Meier survival curves through Engauge Digitizer version 9.8 to extract the data according to the method from the former literature [34]. This process was conducted by two independent researchers (ZB. Meng and YS Zhang) to avoid the reading deviation. > 1 HR represented a worse prognosis compared with a < 1 HR. Statistical heterogeneity across the eligible studies was inspected by processing the Chi-square test (assessing the *P* value) and calculating the I^2^ statistic. If the I^2^ was greater than 50% and *P*-value was less than 0.05, indicating a significant heterogeneity, a random-effects model (the DerSimonian-Laird method) was used. Otherwise the fixed-effects (Mante-Haenszel method) was applied to analyze the pooled HRs.

The sensitivity analyses were conducted to identify the effect of each study on the pooled result.

### Publication bias assessment

We used funnel plots and Egger's test to evaluate the publication bias. All *P* values were two-tailed and P was identified statistically significant if its value < 0.05. Graphical funnel plots were employed to qualitatively exhibit publication bias. And Egger's test was used to demonstrate quantitatively.

## SUPPLEMENTARY MATERIALS AND TABLES





## Abbreviations

OS: overall survival
DFS: disease free survival
HR: hazard ratio
RR: rate ratio
OR: odds ratio
CI: confidence intervals
NOS: Newcastle-Ottawa Quality Assessment Scale
P_S_: P for subgroup difference
RCC: renal cell carcinoma
OSCC: oral squamous cell carcinoma
GC: gastric cancer
PC: pancreatic cancer
IHC: immunohistochemistry
ELISA: enzyme-linked immunosorbent assay
SC: survival curve
