# Correction: B7-H4 as an independent prognostic indicator of cancer patients: a meta-analysis

**DOI:** 10.18632/oncotarget.24571

**Published:** 2018-02-26

**Authors:** Zibo Meng, Feiyang Wang, Yushun Zhang, Shoukang Li, Heshui Wu

**Affiliations:** ^1^ Department of Pancreatic Surgery, Union Hospital, Tongji Medical College, Huazhong University of Science and Technology, Wuhan 430022, China

**This article has been corrected:** The proper name of the institution is as follows:

^1^ Department of Pancreatic Surgery, Union Hospital, Tongji Medical College, Huazhong University of Science and Technology, Wuhan 430022, China

Original article: Oncotarget. 2017; 8:68825-68836. https://doi.org/10.18632/oncotarget.18566

