# Supplementary material for: B7-H4 as an independent prognostic indicator of cancer patients: a meta-analysis
Source: Oncotarget. 2017 Jun 19;8(40):68825–36. doi: 10.18632/oncotarget.18566 (PMC5620299; doi:10.18632/oncotarget.18566)
Supplement: Supplementary file 1 [file oncotarget-08-68825-s001.pdf]

B7-H4 as an independent prognostic indicator of cancer patients: a meta-analysis

Supplementary Materials

Supplementary Table 1: Quality assessment

		Selection				Comparability	Outcome			
name	year	Representativeness of the exposed cohort	Selection of the non-exposed cohort	Ascertainment of exposure	Demonstration that outcome of interest was not present at start of study	Comparability of cohorts on the basis of the design or analysis	Assessment of outcome	Was follow-up long enough for outcomes to occur	Adequacy of follow up of cohorts	total
Liu	2014	0	1	1	1	1	1	1	1	7
Wu	2016	1	1	1	1	2	1	1	1	9
Wang	2016	1	1	1	1	1	1	1	1	8
Wang	2015	1	1	1	1	2	1	1	1	9
Tsiaousidou	2015	0	1	1	1	1	1	1	1	7
Huang	2016	1	1	1	1	2	1	1	1	9
Fukuda	2016	1	1	1	1	2	1	1	1	9
Zhang	2015	0	1	1	1	2	1	1	1	8
Zhang	2015	1	1	1	1	2	1	0	1	8
Dong	2015	1	1	1	1	2	1	1	1	8
Shi	2014	1	1	1	1	2	1	1	1	9
Liang	2013	0	1	1	1	1	1	1	1	7
Fan	2014	1	1	1	1	2	1	1	1	8
Chen	2014	1	1	1	1	2	1	1	1	9
Zhu	2013	1	1	1	1	2	1	0	1	8
Arigami	2011	1	1	1	1	2	1	1	1	9
Maskey	2014	1	1	1	1	1	1	0	1	7
Li	2013	1	1	1	1	1	1	1	1	8
Quandt	2011	0	1	1	1	1	1	1	1	7
Jung	2011	1	1	1	1	1	1	1	1	8
Chen	2011	1	1	1	1	2	1	1	1	9
Jiang	2010	1	1	1	1	1	1	1	1	8
Oikonomopoulou	2008	0	1	1	1	2	1	0	1	7
Zang	2007	1	1	1	1	2	1	1	1	9
Krambeck	2006	1	1	1	1	1	1	1	1	8
Qian	2016	1	1	1	1	1	1	0	1	7
Xu	2016	1	1	1	1	1	1	1	1	7

Supplementary Table 2: Supplementary information included in the literature. See Supplementary_Table_2
