# Supplementary material for: B7-H4 as an independent prognostic indicator of cancer patients: a meta-analysis
Source: Oncotarget. 2017 Jun 19;8(40):68825–36. doi: 10.18632/oncotarget.18566 (PMC5620299; doi:10.18632/oncotarget.18566)
Supplement: Supplementary file 2 [file oncotarget-08-68825-s002.docx]

| **Study** | **Year** | **Country** | **Male/female** | **Follow-up**  **(months)** | **Cut-off value** | **TNM stage** |
| --- | --- | --- | --- | --- | --- | --- |
| Krambeck | 2006 | USA | 174/85 | >60 months | 5% of cells stained | 137/30/51/41(I/II/III/IV) |
| Wang | 2016 | China | 160/156 | 3–80 months | 28.08 ng/ml | 136/180(I-II/III-IV) |
| Simon | 2007 | Italy | 0/233 | <150 months | 426 pg/mg | NR |
| Liang | 2013 | China | 90/95 | >60 months | Stained area: <10% of tumor cells: 1, 10-50%: 2, >50%:3; intensity of staining: absent 0, weak: 1, moderate: 2, strong: 3. Each section had a final grade by multiplying the intensity and area scores. Positive: final score≥4 | 66/119(I+II/III+IV) |
| Fukuda | 2016 | Japan | 109/72 | 3-100 months | 0.383ng/ml | NR |
| Wu | 2016 | China | NR | 1-60 months | H-score^a^ >88.12 | NR |
| Jiang | 2010 | China | 122/34 | >60 months | Stained area: <5% of tumor cells: 0, 6-25%: 1, 26-50%: 2, 51-75%:3, >75%: 4; intensity of staining: absent 0, weak: 1, moderate: 2, strong: 3. Each section had a final grade by multiplying the intensity and area scores. Positive: final score≥9 | 14/22/102/18(I/II/III/IV) |
| Shi | 2014 | China | 97/35 | 1-61 months | 16.85ng/mL | 69/63(I+II/III+IV) |
| Arigami | 2011 | Japan | 74/46 | 1–112 months | B7-H4 immunoreactivity was classified into four groups: negative immunoreaction (-), weak immunoreaction (+), moderate immunoreaction (++), and strong immunoreaction (+++). Positive: moderate or strong B7-H4 immunoreactivity | 62/58(I+II/III+IV) |
| Zhang | 2015 | China | 68/48 | 1-60 months | 70.55ng/mL | 56/60(I+II/III+IV) |
| Chen | 2011 | China | 80/32 | >60months | H-score^a^ >160 | 13/63/20/16 (I/II/III/IV) |
| Huang | 2016 | China | 0/108 | >60 months | Stained area: 1-25% of tumor cells: 1, 26-50%: 2, 51-75%:3, >75%: 4; intensity of staining: absent 0, weak: 1, moderate: 2, strong: 3. Each section had a final grade by multiplying the intensity and area scores. Positive: final score≥3 | 55/51/2(I/II/III) |
| Dong | 2015 | China | 54/50(tissue)  49/37(blood) | 1-60 months | For tissue: Stained area: <10% of tumor cells: 1, 10-50%: 2, >50%:3; intensity of staining: absent 0, weak: 1, moderate: 2, strong: 3. Each section had a final grade by multiplying the intensity and area scores. Positive: final score≥4  For blood: 92.28ng/mL | 62/42(I+II/III)(tissue)  64/22(I+II/III)(blood) |
| Liu | 2014 | Japan | 0/102 | 4.8‑169 months | The intensity of immunostaining for B7-H4 was scored as 0 (negative), 1 (weak), 2 (medium) and 3 (strong).Positive: score>0 | 73/29(I-IIA/IIB-IV) |
| Oikonomopoulou | 2008 | Canada | 0/98 | 2-36 months | >Median value | 14/5/73/6(I/II/III/IV) |
| Zhang | 2015 | China | 72/21 | 5-45 months | 49.12 ng/ml | 56/20/17(I/II/III) |
| Wang | 2015 | China | 38/28 | 1-60 months | Stained area: ≤33 % of tumor cells: 1, 34-66%: 2, >66%: 3; intensity of staining: absent/weak: 1, moderate: 2, strong: 3. Each section had a final grade by multiplying the intensity and area scores. Positive: final score>3 | 43/23(I-II/III-IV) |
| Zhu | 2013 | China | 25/39 | 1-50 months | Stained area: 1-10% of tumor cells: 1, 11-33%: 2, 34-66%:3, >67%: 4; intensity of staining: absent 0, weak: 1, moderate: 2, strong: 3. Each section had a final grade by multiplying the intensity and area scores. Positive: final score>6 | 30/34(I/II+III+IV) |
| Chen | 2014 | China | 41/22 | 1-33 months | ≥30% cells stained | NR |
| Fan | 2014 | China | 48/14 | 1-60 months | Stained area: <5% of tumor cells: 0, 6-25% of tumor cells: 1, 26-50%: 2, 51-75%:3, >75%: 4; intensity of staining: absent 0, weak: 1, moderate: 2, strong: 3. Each section had a final grade by multiplying the intensity and area scores. Positive: final score≥4 | 11/51(Superfcial/invasive) |
| Maskey | 2014 | China | 35/21 | 12-52 months | Stained area: <5% of tumor cells: 0, 6-25% of tumor cells: 1, 26-50%: 2, 51-75%:3, >75%: 4; intensity of staining: absent 0, weak: 1, moderate: 2, strong: 3. Each section had a final grade by multiplying the intensity and area scores. Positive: final score≥9 | 12/44(I–II/III–IV) |
| Li | 2013 | China | 37/12 | 1-120 months | Stained area: <5% of tumor cells: 0, 6-25% of tumor cells: 1, 26-50%: 2, 51-75%:3, >75%: 4; intensity of staining: absent 0, weak: 1, moderate: 2, strong: 3. Each section had a final grade by multiplying the intensity and area scores. Positive: final score≥9 | 13/29/7(I/II/III) |
| Tsiaousidou | 2015 | Greece | 21/20 | 2–31 months | >10 % cells stained | 4/35/2(I/II/III) |
| Quandt | 2011 | Germany | 16/13 | >60 months | Stained area: <25 % of tumor cells: 0, 25-75%: 1, >75%: 2; intensity of staining: absent: 0, weak: 1, moderate: 2, strong: 3. Each section had a final grade by multiplying the intensity and area scores. IRS≥4 | 26/3(III/IV) |
| Zang | 2007 | USA | 823/0 | Median 84 months | Intensity of staining was classified as absent, weak, moderate, strong. Positive: strong. | NR |
| Jung | 2011 | Korea | 39/63 | 6-84 months | ≥10% of cells stained | NR |
| Qian | 2016 | China | 26/17 | 1-25 months | Stained area: <5% of tumor cells: 0, 6-25% of tumor cells: 1, 26-50%: 2, 51-75%:3, >75%: 4; intensity of staining: absent 0, weak: 1, moderate: 2, strong: 3. Each section had a final grade by multiplying the intensity and area scores. Positive: final score≥9 | NR |
| Xu | 2016 | China | 26/14 | 12-134 months | ≥10% cells stained | 11/15/8/6 |

^a^ H-score = (% tumor cells unstained * 0) + (% tumor cells stained weak * 1) + (% tumor cells stained moderate * 2) + (% tumor cells stained strong * 3). The

H-scores ranged from 0 (100% negative tumor cells) to 300 (100% strong staining tumor cells).
